# The effects of humidity on thermoregulatory physiology of a small songbird

**DOI:** 10.1242/jeb.247357

**Published:** 2024-07-02

**Authors:** Cody K. Porter, Kevin M. Cortes, Ofir Levy, Eric A. Riddell

**Affiliations:** ^1^ Department of Ecology, Evolution, and Organismal Biology, Iowa State University, Ames, IA 50011, USA; ^2^ Faculty of Life Sciences, School of Zoology, Tel Aviv University, Tel Aviv 6997801, Israel; ^3^ Department of Biology, University of North Carolina – Chapel Hill, Chapel Hill, NC 27599, USA

**Keywords:** Body temperature, Evaporative water loss, Humidity, Metabolic rate, Scholander–Irving model, Thermoregulation, Tree swallow

## Abstract

Scholander–Irving curves describe the relationship between ambient temperature and metabolic rate and are fundamental to understanding the energetic demands of homeothermy. However, Scholander–Irving curves are typically measured in dry air, which is not representative of the humidity many organisms experience in nature. Consequently, it is unclear (1) whether Scholander–Irving curves (especially below thermoneutrality) are altered by humidity, given the effects of humidity on thermal properties of air, and (2) whether physiological responses associated with Scholander–Irving curves in the lab reflect organismal performance in humid field conditions. We used laboratory experiments and biophysical models to test the effects of humidity on the thermoregulatory physiology of tree swallows (*Tachycineta bicolor*). We also tested whether physiological responses measured under lab conditions were correlated with field body temperatures and nestling provisioning rates. We found that humidity reduced rates of evaporative water loss but did not have large effects on body temperature or metabolic rate, suggesting that swallows can decouple evaporative cooling, body temperature and metabolic rate. Although the effect of humidity on metabolic rate in the lab was small, our biophysical models indicated that energetic costs of thermoregulation were ∼8% greater in simulations that used metabolic rates from birds in humid compared with dry conditions. Finally, we found mixed evidence that physiological responses measured in the lab under humid or dry conditions were associated with body temperature and nest provisioning rates in the field. Our results help clarify the effect of humidity on endotherm thermoregulation, which may help forecast organismal responses to environmental change.

## Teaser

**Highlighted Article:** Temperature and humidity interact in complex ways to shape the thermoregulatory responses of tree swallows (*Tachycineta bicolor*).

## INTRODUCTION

A central challenge for endotherms is the energetic demand associated with maintaining high, relatively stable body temperatures under variable environmental temperatures (Bennett and Ruben, 1979). The classic framework for understanding these challenges is the Scholander–Irving model of thermoregulation (Scholander et al., 1950). Over a range of ambient temperatures termed the ‘thermoneutral zone’ (TNZ), heat balance is maintained without energy expenditure exceeding an organism's basal metabolic rate (i.e. the metabolic rate of inactive, normothermic, postabsorptive, non-reproductive adults; McNab, 1997). As ambient temperatures increase, a point is reached at which metabolic rate begins to increase (the ‘upper critical temperature’; UCT) as organisms engage in costly cooling responses such as panting (Withers et al., 2016). Similarly, as ambient temperatures decrease below the TNZ, metabolic rate begins to increase (at the ‘lower critical temperature’; LCT), as organisms generate metabolic heat to compensate for heat loss and maintain a stable body temperature (Withers et al., 2016). Therefore, Scholander–Irving curves encompass key parameters that describe an organism's physiological responses to environmental temperatures. However, relatively little is known about the effects of humidity on Scholander–Irving curves and the consequences for organismal performance, in part because Scholander–Irving curves are typically measured under dry or unreported humidity conditions in the lab (e.g. Muñoz-Garcia et al., 2016; Smith et al., 2017; Ramirez et al., 2022).

**List of symbols and abbreviations**

EHL: rate of evaporative heat loss
EWL: rate of evaporative water loss
LCT: lower critical temperature
MHP: rate of metabolic heat production
PIT: passive integrated transponder
RFID: radio frequency identification
*T*_b_: body temperature
*T*_es_: standard operative temperature
TNZ: thermoneutral zone
UCT: upper critical temperature
*V*_CO_2__: rate of carbon dioxide production

Humidity might influence endothermic metabolic rates by changing the potential for evaporative water loss and/or the thermal properties of air. For example, at warm temperatures, high humidity inhibits evaporative water loss (Lasiewski et al., 1966; Powers, 1992; Gerson et al., 2014; van Dyk et al., 2019), thereby increasing the risk of hyperthermia (Gerson et al., 2014; van Dyk et al., 2019) and metabolic expenditure above the UCT (van Dyk et al., 2019). The effects of humidity on metabolic rate within and below the TNZ are less clear. From a purely physical perspective, evaporative water loss (and thus evaporative cooling) should be elevated under dry conditions because of a higher vapor pressure deficit, the physical driver of evaporation rates (Campbell and Norman, 1998; Withers et al., 2016). High evaporative water loss under dry conditions could exacerbate heat loss at low temperatures (Edwards and Haines, 1978), thus resulting in lower body temperatures or increasing the need for metabolic heat production. Alternatively, heat loss and metabolic responses to low temperatures may be greater under humid conditions because humidity increases the thermal conductivity and specific heat capacity of air, potentially increasing the rate of heat transfer away from the body and the amount of heat needed to reach thermal equilibrium, respectively (Crow, 1988; Denny, 1993). To date, the evidence bearing on these alternative hypotheses is mixed, with some studies finding that humidity increases metabolic rate (Cooper et al., 2020; Gilson et al., 2021), decreases metabolic rate (Cooper and Withers, 2008) or has no effect (Edwards and Haines, 1978; Webster and King, 1987; Powers, 1992; Klüg-Baerwald and Brigham, 2017; Eto et al., 2017). Therefore, testing the effects of temperature and humidity on thermoregulatory physiology will improve our ability to predict metabolic expenditure in nature, where both environmental variables vary across space and time.

If humidity affects endotherm thermoregulation, laboratory experiments that ignore humidity may not fully capture variation in thermoregulatory performance in the field, especially for endotherms inhabiting humid environments. Addressing the effects of humidity represents a considerable challenge for physiological ecologists because Scholander–Irving curves are used to predict organismal performance and fitness in nature (Fristoe et al., 2015) and forecast responses to climate change (Araújo et al., 2013; Khaliq et al., 2014). One possible solution could be to measure thermoregulatory performance under humid air conditions in the lab to determine whether these measures reflect thermoregulatory performance (or even performance in general) in more realistic environmental conditions compared with experiments conducted in completely dry air. Few studies have related physiological responses of individuals in the lab to individual performance in the field (but see Gilbert and Miles, 2017), and we are not aware of any that have tested whether individual variation in thermoregulatory performance in endotherms in the lab reflects performance in the field. Testing the correlation between lab- and field-based physiological responses will help to determine the ecological relevance of laboratory experiments, particularly by including the effects of humidity.

Here, we studied the effects of humidity and temperature on thermoregulatory performance in tree swallows (*Tachycineta bicolor*) using a combination of laboratory experiments, biophysical modeling and field data on performance. We focus on the physiological consequences of humidity under low ambient temperatures (15–32°C), given that cold, wet conditions are common during the breeding season at our study site and are well known to cause mass mortality and reproductive failure in tree swallows (Griebel and Dawson, 2018; Cox et al., 2019; Shipley et al., 2020; Garrett et al., 2022). We conducted laboratory experiments to measure the effects of humidity on the relationships between ambient temperature and the volume of carbon dioxide production (a proxy for metabolic rate), body temperature and evaporative water loss within and below the TNZ. Next, we used a biophysical model (parameterized by our laboratory experiments, local weather data and biophysical properties of tree swallows) to explore the consequences of humidity variation for resting energetic demands in the field. Finally, we tested whether individual variation in thermoregulatory traits measured in the lab under humid or dry conditions were associated with field active body temperatures or nest provisioning rates in the field. Together, these data help to reveal the effects of humidity on endotherm energetics; the extent of coupling between evaporative water loss, body temperature and metabolic rate; and the ecological relevance of laboratory experiments.

## MATERIALS AND METHODS

### Study site

We conducted this study on tree swallows [*Tachycineta bicolor* (Vieillot 1808)] between May and July 2021 at the Iowa State University (ISU) Horticulture Research Farm (latitude: 42.106328, longitude: −93.589807), as part of a long-term nest box study that began in 2003 (Vleck et al., 2012). Tree swallows occupy nest boxes located throughout the farm that are spaced 10–20 m apart in grids or lines, with a total of ∼138 available nest boxes. All nest boxes are located near agricultural research plots and within 600 m of a small lake.

### Lab methods

All protocols were approved by the Iowa State University Animal Care and Use Committee (IACUC protocol no. 20-169), the Iowa Department of Natural Resources (permit no. SC1428), the United States Fish and Wildlife Service (permit no. MBPER0003052) and the United States Geological Survey Bird Banding Lab (permit no. 07014-AB).

We began capturing adults for experimentation when nestlings were between 10 and 15 days old to minimize nest abandonment. We captured birds between 15:00 and 18:00 h and transported them to an experimental lab space at ISU. We collected 10 males and 16 females for our laboratory experiments, with an average mass of 18.91 g (±1.70 g s.d.). Experiments began between 20:00 and 21:00 h and concluded by 05:00 h the following morning, which allowed us to take measurements of metabolic rate at rest during a period of inactivity (Jimeno et al., 2020). Additionally, tree swallows provision offspring during the day, so measuring birds at any other time might negatively impact nestlings (Lendvai et al., 2015). We did not remove birds from their nests during nights with inclement weather to reduce negative impacts to nestlings. We released experimental birds at their nesting site immediately following experiments so they could resume offspring provisioning the following morning. None of the experimental adults abandoned their nest after the experiments.

We placed birds in a respirometry chamber composed of a locking lid Tritan™ plastic food storage container with a rubber gasket (20×14×12 cm, 1.8 liters, Rubbermaid, Atlanta, GA, USA). We drilled holes into the lid of the chamber and used stoppers with hose barbs to maintain an airtight seal while allowing air to be pumped into and out of the chamber. We placed a mesh grate inside the chamber as a platform for the bird to stand on. The mesh platform also allowed any excrement to fall into a small pool of mineral oil beneath the bird to ensure that water vapor pressure readings were coming only from respiratory and cutaneous water loss. We placed the chamber in a temperature- and humidity-regulating incubator (I-36VL, Percival Scientific, Perry, IA, USA) and used a camera monitoring system, composed of a network video recorder (ZOSI ZR08EN, ZOSI, Zhong Shan City, Guang Dong Province, China) and camera with night vision capabilities (ZOSI 5MP camera model ZG2615D-WS) to livestream and record the bird during measurements.

We assigned experimental birds to either a dry (∼0% relative humidity) or humid (∼60% relative humidity) treatment and each bird was exposed to five different temperatures: three below the TNZ (15, 19 and 23°C) and two within the TNZ (30 and 32°C; Williams, 1988). For the humid treatments, we set the incubator to 60% relative humidity for each temperature. For the dry treatment, we set the incubator to the lowest relative humidity setting and then further dried the air using two parallel columns of Drierite™ to reduce the humidity of the respirometry chamber to near 0% relative humidity (average water vapor pressure=0.004 kPa).

Once the desired temperature and humidity settings were reached for each trial, we allowed birds to acclimate at each temperature for 30 min before data collection began. We exposed each bird to the experimental temperature in a non-consecutive order (19, 15, 23, 32 and 30°C), and this order was used for all birds in both treatments. We selected this order over a fully randomized design due to time constraints and cooling/heating efficiencies of the incubator. To account for potential effects of circadian rhythm on metabolic rate (e.g. Chew et al., 1965), we incorporated time in our statistical analyses (see Statistical analyses). Body temperature data were taken from the passive integrated transponder (PIT) tags every 10 s by a Biomark HPR Plus reader (see ‘Field methods’ section below).

Gas exchange analysis followed a protocol typical of respirometry studies (Whitfield et al., 2015). We zeroed and spanned the water vapor pressure and CO_2_ analyzers prior to the start of the experiments following standard protocols (Lighton, 2008). For both humid and dry treatments, we used a diaphragm pump (Gast Manufacturing Inc., Benton Harbor, MI, USA) paired with a mass flow controller (Alicat Scientific Inc., Tucson, AZ, USA) that maintained a flow rate of 4 liters of air per minute from the incubator housing the chamber into the respirometry chamber. Air passing out of the respirometry chamber was subsampled (250 ml min^−1^) using a gas analyzer (Field Metabolic, Sable Systems International, Las Vegas, NV, USA) that recorded the partial pressure of water vapor pressure and CO_2_. In our experiment, the pump was located before the animal chamber and is thus a push system. This pump pulled air from an empty incubator set to the desired humidity to regulate the amount of water vapor in the system. The pump itself was located in the empty incubator and pushed air into the mass flow controller. We measured the baseline composition of air being pumped into the chamber by using a second line of air pumped in parallel to the analyzer. We manually switched the analyzer between the sample line and the baseline until we obtained a minimum stable measurement of at least 5 min. We identified stable measurements based on the lowest measurement observed without any irregular spikes or changes and when the animal was immobile (as confirmed by video recording). We used the relative differences of the gases between these two lines to calculate evaporative water loss (EWL) and the volume of CO_2_ production (*V*_CO_2__) following standard protocols in Riddell et al. (2017) and Lighton (2008), respectively.

We also calculated vapor pressure deficit (VPD; kPa) using ambient temperature and relative humidity (RH). We calculated VPD as:
(1)

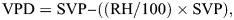
where SVP is the saturation vapor pressure (kPa). VPD is a measure of humidity that describes the drying power of air while controlling for the effect of temperature, unlike relative humidity alone (Anderson, 1936).

Designing experiments to evaluate the effects of humidity and temperature is challenging for several reasons. Ideally, experiments control the humidity such that the same VPD (i.e. evaporative demand) is maintained at each temperature. However, across a broad range of temperatures (as in our study), this becomes impossible because cool temperatures can only hold a small fraction of water vapor compared with hot temperatures. For instance, the VPD of completely dry air at 10°C is 1.22 kPa (0% relative humidity), but maintaining the same VPD at 32°C would correspond to 74.3% relative humidity. To our knowledge, every study on avian water loss confounds temperature and VPD (i.e. animals experience a unique combination of temperature and VPD). We used weather station data from 2004 to 2022 to determine whether temperature and VPD were correlated at our field site. We found that temperature and VPD are generally positively and non-linearly correlated (Fig. S4); however, tree swallows experience a wide range of VPDs at almost every temperature, from nearly completely dry to completely saturated. Thus, designing experiments with a balanced temperatures and VPDs is ecologically relevant and may inform how tree swallows independently respond to both environmental variables.

Water loss experiments also need to ensure that the desired VPD is experienced. In our study, we maintained the same wind speed to ensure that convective conditions did not confound the experimental design. One potential drawback of this approach is that EWL from animals could increase the humidity of chamber air relative to the incurrent air (e.g. Lasiewski et al., 1966). However, the high flow rate and chamber volume we used largely negated this issue, given that incurrent and excurrent humidity and VPD were very similar (Table S1). We also decided to treat humidity as a factor (either dry or humid) to evaluate the effect of temperature in these two contrasting hydric conditions. Though imperfect, we propose this design balances the statistical issues related to experiments on temperature and humidity with the ability to answer the physiological question at hand.

### Biophysical model

We used a previously developed model of endotherm thermoregulation (Riddell et al., 2019) to explore the effect of humidity on the energetic demands of tree swallows at our field site. This model simulates heat balance using the morphological characteristics of birds in a complex radiative and thermal environment. We estimated: (1) body shape; (2) average feather length across the dorsum and ventrum; (3) plumage depth across the dorsum and ventrum; and (4) feather reflectance of the dorsum and ventrum for adult tree swallows in our population. We assumed an average body shape, feather length and plumage depth based upon previously developed allometric relationships between each of these traits and body mass (Riddell et al., 2019). We used an empirical estimate of body mass (18.9 g) based on the mass of our experimental tree swallows. For body temperature, we assumed a value of 39°C at night (when the sun was below the horizon) and a value of 43°C during the day. These temperatures were based upon the daily variation in body temperature measured in field-active individuals (Fig. S1; see Field methods below).

We measured dorsal and ventral feather reflectance every 1 nm from 350 to 2002 nm of 27 females using an Ocean Insight DH-2000-BAL deuterium–halogen light source connected to an Ocean Insight Ocean HDX spectrometer and an Ocean Insight NIRQuest near-infrared spectrometer (Ocean Insight, Orlando, FL, USA). Prior to each series of measurements, we used a Spectralon™ white standard to standardize spectrometer readings. We measured feather reflectance at a standardized distance (2 cm) from the feather surface with a 45 deg angle between the light source and fiber optic cable. The angle, distance and area of measurement were standardized using an RPH-1 reflection probe holder (Ocean Optics, Largo, FL, USA). We measured reflectance from three feather patches on the dorsum (crown, mantle and rump) and one on the ventrum (breast) of each individual. We averaged across dorsal patches to estimate overall dorsal reflectance. We also corrected reflectance curves for solar radiation using the ASTM G-172 standard irradiance spectrum for dry air provided by SMARTs version 2.9.2. We calculated the solar-corrected reflectance value of the dorsum and ventrum by multiplying the intensity of solar radiation by the empirical reflectance, integrating across all wavelengths, and dividing by the total intensity of solar radiation (Gates, 1980). We averaged solar absorptance (1–reflectance) of the dorsum and ventrum across all individuals and incorporated these values into our biophysical model (see Riddell et al., 2019 for more details on the integration of biophysical traits into this model).

To estimate the effect of humidity on the resting metabolic demands of tree swallows in the field, we incorporated the regressions between ambient temperature and *V*_CO_2__ from our laboratory experiments under humid and dry conditions into the biophysical model. Specifically, we estimated *V*_CO_2__ based on the standard operative temperature (*T*_es_) of tree swallows, which we estimated by combining biophysical simulations with local weather station data. *T*_es_ is a metric that incorporates the effects of air temperature, solar radiation, wind speed, body size, and the interactions between wind speed and insulation on heat balance of endotherms (Riddell et al., 2023). More importantly, *T*_es_ can be used to directly relate the results from laboratory experiments to the thermal environment that organisms experience in nature (Campbell and Norman, 1998). We estimated resting *V*_CO_2__ for each hour of the day based on the simulated *T*_es_ and the model estimates for dry and humid conditions from the laboratory experiments. We included regressions above and below the LCT for both dry and humid treatments using breakpoint regressions (see Statistical analyses). We also incorporated variability of *V*_CO_2__ into the simulations based upon the average standard deviation in *V*_CO_2__ below and above the LCT under dry and humid conditions. We specifically used the random.normal() function in the NumPy module to estimate *V*_CO_2__ (assuming a normal distribution) based on the average *V*_CO_2__ (at the particular *T*_es_) and the standard deviation (if either above or below the LCT for both dry and humid treatments). We ran these analyses over 9 years (2014–2023) of hourly weather data from a weather station located at our field site (station AEE14: https://mesonet.agron.iastate.edu/agclimate/hist/hourly.php). We restricted analyses to the breeding season of our tree swallow population (1 April–31 July) and the range of experimental temperatures we studied (15–32°C), which corresponded to 65% of hourly weather observations across the nine breeding seasons. These simulations do not include the effects of activity on metabolic expenditure, and thus only represent resting costs of tree swallows.

### Field methods

We began monitoring nest boxes in May 2021 and continued until the last tree swallow departed in July. We checked nests every 3 days for nest materials and eggs/nestlings. Tree swallows lay one egg each day, so we back-calculated lay dates if eggs were laid on days when nests were not checked (Winkler et al., 2013). We used predicted hatch dates (7 days after final egg laid) to check for the presence of nestlings and continually checked until hatch date was confirmed. After nestlings reached day 15, we did not disturb nests to avoid initiating premature fledging (Jimeno et al., 2020).

After 8 days of egg incubation, we used hinged traps to capture adult males and females. Once captured, we banded, weighed and injected adults with a uniquely identifying temperature-sensitive PIT tag (Bio-Therm13, Biomark, Boise, ID, USA). We injected PIT tags into the abdomen by gently pulling the loose abdominal skin away from the bird and inserting the syringe near, but to the side of, the cloaca. This methodology differs from other studies of tree swallow thermoregulation, which injected PIT tags into the nape (Tapper et al., 2020). Nonetheless, our approach is commonly used in studies of songbird thermoregulation (Albright et al., 2017; Czenze et al., 2020) and the body temperatures we documented closely match those recorded in Tapper et al. (2020, mean body temperature=42.0°C; mean body temperature in our study=42.2°C). Although PIT tags injected into the body cavity provide the best estimate of core body temperature, subcutaneously injected PIT tags in smaller birds (<25 g; such as tree swallows) provide nearly identical measurements as PIT tags in the core, likely because of the reduced thermal gradient between the core and peripheral tissue in smaller birds (Oswald et al., 2018). Subcutaneous PIT tags also reduce the risk of injury to small birds (Oswald et al., 2018). Given these comparisons and considerations, subcutaneous measurements likely provide adequate estimates of body temperature.

We also used the PIT tags to measure body temperature and provisioning rates in the field. We collected field body temperature (*T*_b_) and provisioning data with a Biomark™ SSM8 multiplexing radio frequency identification (RFID) antenna system. We equipped the entry point of active nest boxes with RFID antennas that read the PIT tag each time a bird entered the nest to provision offspring. RFID antennas were set to wait 10 s between datalogging for unique individuals, but otherwise, the antennas took continuous readings throughout the entirety of their deployment on the nests. As the maximum reach of the RFID antenna cables was 20 m, we placed the multiplexing unit in positions to maximize the number of nests that could be monitored at one time (generally 1–3 nests). Every 2–4 days we moved the unit to different sets of nests, such that we were able to monitor provisioning at each nest for 1–2 time periods over the course of the breeding season.

### Data processing

We lag- and response-corrected water vapor pressure and raw CO_2_ data using the lag correction and *z*-transformation functions in Expedata (version 1.9.27, Sable Systems International) to keep both gases in phase with one another, accounting for the slight time delay between gas measurements. Relative differences between water vapor pressure and CO_2_ samples and baseline readings were converted to EWL and *V*_CO_2__ in Expedata using eqns 3 and 4 in Riddell et al. (2017) for EWL, and eqns 11.7 and 11.8 in Lighton (2008) for *V*_CO_2__. We also calculated VPD-corrected EWL (EWL/VPD) to assess whether EWL is a physical byproduct of VPD or whether tree swallows regulate EWL with respect to humidity and/or temperature. We defined *V*_CO_2__* *as the average of the lowest 1-min interval within a stable sampling period (Whitfield et al., 2015). We defined EWL by either taking the average of the most stable 1-min period of the measurement or using a function in Expedata to obtain the asymptote of the reading when EWL response time was slow to stabilize. *T*_b_ measurements during laboratory experiments were imported into Expedata to sync with EWL and *V*_CO_2__ using timestamps taken at each *T*_b_ reading. Values of *T*_b_ were calculated as the average value over the entirety of stable measurement periods (i.e. periods with no sudden spikes in *T*_b_). Finally, we calculated metabolic heat production (MHP) and evaporative heat loss (EHL) using EWL, *V*_CO_2_ _and a respiratory exchange ratio of 0.71, as our birds were all likely post-absorptive (Whitfield et al., 2015; Noakes et al., 2016). This allowed us to calculate the efficiency of evaporative cooling between humidity treatments by calculating the ratio between EHL and MHP (Gerson et al., 2014). This ratio describes the amount of heat loss via evaporation while accounting for increases in MHP generated by organismal responses that increase EHL such as gular fluttering and panting.

We combined field measurements of *T*_b_ in Python using the pandas library (McKinney, 2010) and glob function, resulting in average hourly *T*_b_ measurements for each individual. We calculated hourly provisioning rates using R code from a recent study on tree swallow provisioning rates (Tapper et al., 2020). This code used the R package feedr (LaZerte et al., 2017) to calculate provisioning rates while accounting for the potential of provisioning adults to linger on the nest box after feeding their offspring. We removed the first and last hours of data for each individual to account for uncertainty in the exact time the multiplexing unit was moved.

### Statistical analyses

We performed all statistical analyses in R (version 4.3.1; https://www.r-project.org/). We used linear mixed effects models and type II ANCOVAs in the lme4 and lmerTEST packages to test whether humidity altered the relationship between temperature and thermoregulatory traits (*V*_CO_2__, EWL, VPD-corrected EWL, *T*_b_ and EHL/MHP). We used each of these thermoregulatory traits as the response variable in separate models. We ran separate models for *V*_CO_2__ below and within the TNZ. Temperature, humidity and their interaction were our predictor variables of interest, though we also included mass as a covariate and sex as a factor. We included individual and hour of measurement as random effects. We treated temperature as a factor in analyses of EWL, VPD-corrected EWL, *T*_b_ and EHL/MHP because we did not have strong *a priori* expectations for linear responses as we did for *V*_CO_2_ _within and below the TNZ. We used Tukey’s honest significant difference (HSD) *post hoc* comparisons in the emmeans package to conduct pairwise comparisons of EWL, *T*_b_ and EHL/MHP. We compared the distribution of hourly resting *V*_CO_2__ values simulated under humid and dry conditions with our biophysical model using a paired Wilcoxon signed rank test.

In addition to comparing *V*_CO_2_ _across temperature and humidity treatments, we were also interested in testing whether humidity affects the LCT and the slope of the relationship between temperature and *V*_CO_2_ _below the TNZ (a measure of the temperature sensitivity of metabolic rate). To estimate these parameters, we used Bayesian breakpoint regressions in the mcp R package (https://CRAN.R-project.org/package=mcp). We ran separate models for humid and dry conditions, with *V*_CO_2_ _as the response variable, ambient temperature as a predictor variable, and individual identity as a random slope. We did not include mass or sex in this model because we found no evidence that either affected *V*_CO_2_ _(see Results).

Finally, we used linear models to test whether thermoregulatory traits measured under humid or dry conditions in the lab were correlated with individual variation in *T*_b_ or nest provisioning rate (number of offspring provisioning trips per hour) collected from the same individuals in the field. Tree swallows exhibit repeatable individual variation in field *T*_b_ (Tapper et al., 2021), but correlations between lab and field physiology have yet to be explored. We ran separate models testing whether average *T*_b_ in the lab is correlated with average *T*_b_ in the field and whether *T*_b_ variability (i.e. standard deviation of *T*_b_) in the lab is correlated with *T*_b_ variability in the field. We also ran separate models testing whether nest provisioning rate is correlated with two aspects of metabolic rate in the lab: (1) average *V*_CO_2_ _within the TNZ and (2) the temperature sensitivity of metabolic rate. Because we ran two models with provisioning rate as the response variable, we applied a Benjamini–Hochberg correction to *P*-values from these analyses. We hypothesized that individuals with higher *V*_CO_2_ _would have a greater physiological capacity to provision young (Nilsson, 2002). We also hypothesized that individuals with more temperature-sensitive metabolic rate would provision young at higher rates under the variable weather conditions at our field site (e.g. Norin and Metcalfe, 2019). We included an interaction between the physiological trait and lab humidity treatment in all models, which also allowed us to test our hypothesis that physiological traits under humid lab conditions would better reflect physiology in the field than the same traits measured under dry lab conditions. We also included average hourly air temperature (*T*_a_) during field measurements and standard deviation in hourly *T*_a_ during field measurements as predictor variables to control for variation in the field temperatures individuals experienced, as not all individuals were measured over the same time frame in the field. Our models did not include sex and body mass, given that none of our other analyses found significant effects of these variables. For all lab-to-field comparisons, we limited our dataset to field *T*_a_ that were within the experimental temperature range we used in the lab (15–32°C; 97% of our field temperature measurements fell within this range).

## RESULTS

### Lab results

#### *V*_CO_2__

**Fig. 1.**
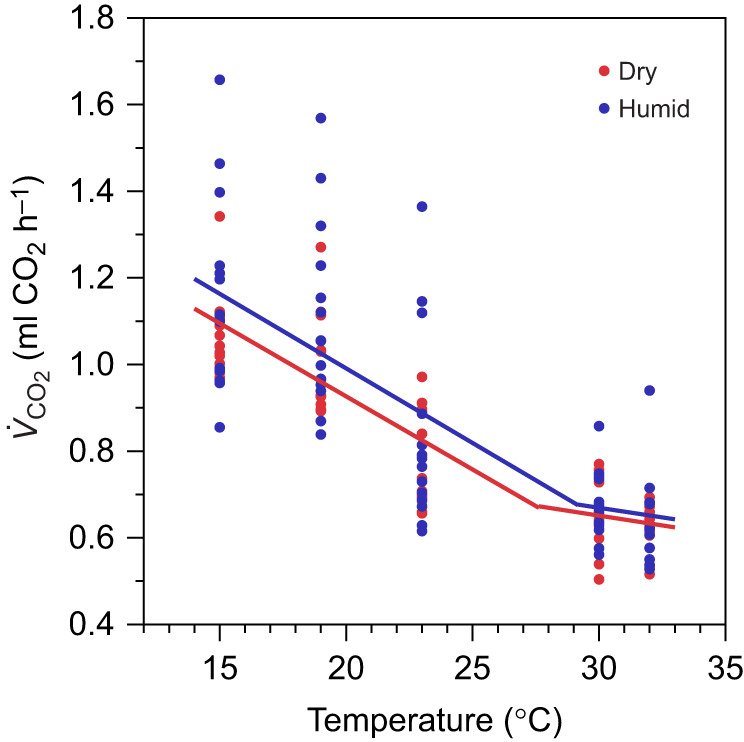
**Below the thermoneutral zone (TNZ), *V*_CO_2__ significantly increased as temperature decreased under both dry (0% relative humidity; red) and humid conditions (60% relative humidity; blue).** Within the TNZ (30–32°C), there was no relationship between temperature and *V*_CO_2__. We also did not find statistical differences in *V*_CO_2__, lower critical temperature or the temperature sensitivity of *V*_CO_2_ _below thermoneutrality. Circles represent raw data from individual birds. Solid lines represent Bayesian breakpoint regressions.

We found similar resting *V*_CO_2_ _to previous experimental work on tree swallows under similar conditions (mean±s.d. resting *V*_CO_2__ at 30°C from Zhang et al., 2021: 0.881±0.133 ml O_2_ min^−1^; mean value at 30°C in our study: 0.874±0.187 ml O_2_ min^−1^). Below the TNZ (15–23°C), we found a pattern typical of Scholander–Irving curves, where *V*_CO_2_ _increased as temperature decreased (*F*_1,171_=236.82, *P*<0.001; Fig. 1). Within the TNZ (30–32°C), we found no relationship between *V*_CO_2_ _and temperature (*P*=0.108; Fig. 1), as expected. Within the TNZ, we found no evidence that humidity treatment (*P*=0.961), mass (*P*=0.574), sex (*P*=0.564), or the interaction between temperature and humidity (*P*=0.981) affected *V*_CO_2__. Likewise, we found no evidence that humidity treatment (*P*=0.396), mass (*P*=0.104), sex (*P*=0.073), or the interaction between temperature and humidity (*P*=0.840) affected *V*_CO_2_ _below the TNZ. Though not statistically significant, *V*_CO_2_ _was 2.0–10.2% higher for tree swallows exposed to humid conditions compared with dry conditions (15°C=7.9%, 19°C=10.2%, 23°C=7.3%, 30°C=2.4%, 32°C=2.0%). *V*_CO_2_ _was also more variable under humid conditions relative to dry conditions (dry s.d. on average *V*_CO_2__=0.057; humid s.d.=0.060).

#### LCT and thermal sensitivity of *V*_CO_2__

Consistent with the above *V*_CO_2_ _analysis based on linear regressions, our breakpoint regression analysis also found no evidence that the thermal sensitivity of *V*_CO_2_ _differed between dry conditions (average±95% CI=−0.038±0.006) and humid conditions (average±95% CI=−0.040±0.004). We also found no evidence that LCT differed between dry conditions (average±95% CI=27.6±2.9°C) and humid conditions (average±95% CI=29.2±1.1°C).

#### EWL and VPD-corrected EWL

**Fig. 2.**
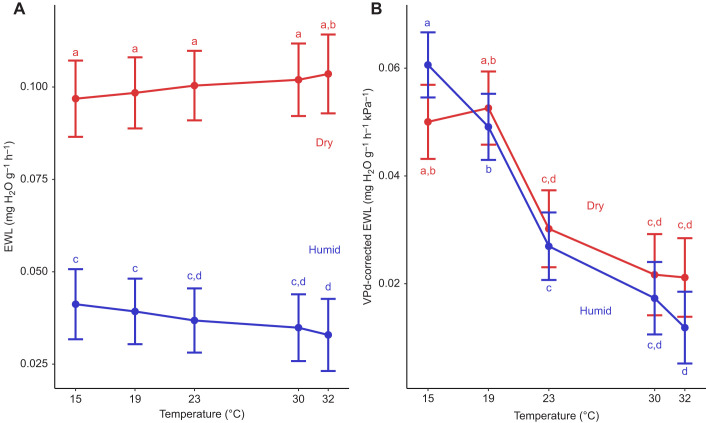
**Relationship between evaporative water loss (EWL) and humidity and temperature.** (A) Rates of EWL were higher under dry conditions (0% relative humidity; red line) than under humid conditions (60% relative humidity; blue line) across all temperatures. Within the dry treatment, there was no consistent relationship between EWL and temperature. Within the humid treatment, EWL tended to decrease as temperatures increased. (B) EWL corrected for vapor pressure deficit (VPD) decreased as temperature increased under both dry conditions and humid conditions. Note that a purely physical model of EWL predicts no relationship between VPD-corrected EWL and humidity or temperature. VPD-corrected EWL did not differ between humidity treatments at any temperature. Different letters denote significant differences based on Tukey HSD *post hoc* comparisons. Error bars are 95% confidence intervals.

We found higher rates of EWL in dry conditions compared with humid conditions (*F*_1,22_=105.59, *P*<0.001; Fig. 2A). We also found that temperature (*F*_4,24_=8.82, *P*<0.001) and its interaction with humidity (*F*_4,238_=12.64, *P*<0.001) affected EWL. In dry conditions, EWL was relatively uniform across temperatures (Fig. 2A). In humid conditions, we found evidence for a slight reduction in EWL as temperatures increased (Fig. 2A), with significantly lower EWL at 32°C than both 15°C (*P*=0.010) and 19°C (*P*=0.006). We found no evidence that mass (*P*=0.993) or sex (*P*=0.404) affected EWL.

Contrary to purely physical expectations of EWL, we found a negative relationship between VPD-corrected EWL and temperature (*F*_4,14_=328.42, *P*<0.001; Fig. 2B). We also found that the interaction between temperature and humidity affected VPD-corrected EWL (*F*_4,245_=7.71, *P*<0.001). However, we found no significant pairwise differences in VPD-corrected EWL between humid and dry conditions at any temperature (*P*≥0.299 for all comparisons), consistent with physical expectations. We found no evidence that humidity alone, mass, or sex affected VPD-corrected EWL (*P*≥0.196 for all comparisons).

#### *T*_b_

**Fig. 3.**
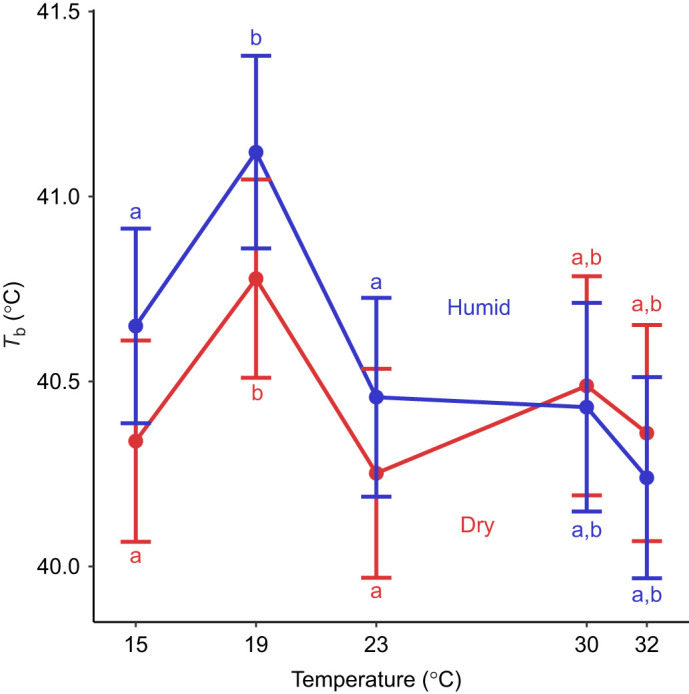
**Body temperature (*T*_b_) was not affected by ambient temperature under dry conditions (0% relative humidity; red line) or humid conditions (60% relative humidity; blue line).** *T*_b_ also did not differ between humidity treatments at any temperature. Different letters denote significant differences based on a Tukey HSD *post hoc* comparison. Error bars are 95% confidence intervals.

We found that temperature (*F*_4,15_=19.16, *P*<0.001) and its interaction with humidity (*F*_4,227_=4.21, *P*=0.003) affected *T*_b_ (Fig. 3). However, we found no significant pairwise differences in *T*_b_ between humid and dry conditions at any temperature (*P*≥0.681 for all comparisons). We found no evidence that humidity alone (*P*=0.306), mass (*P*=0.693) or sex (*P*=0.133) affected *T*_b_.

#### Efficiency of evaporative cooling (EHL/MHP)

**Fig. 4.**
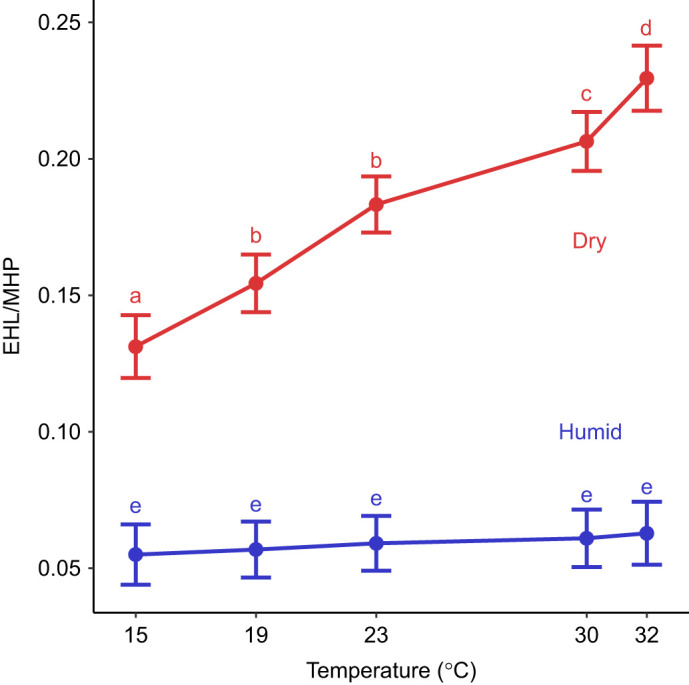
**The efficiency of evaporative cooling (EHL/MHP) was greater under dry conditions (0% relative humidity; red line) than under humid conditions (60% relative humidity; blue line) across all temperatures.** Within the dry treatment, EHL/MHP increased as temperatures increased. Within the humid treatment, there was no relationship between EHL/MHP and temperature. Different letters denote significant differences based on a Tukey HSD *post hoc* comparison. Error bars are 95% confidence intervals.

We found that EHL/MHP was greatest under dry conditions (*F*_1,20_=248.38, *P*<0.001) and high temperatures (*F*_4,20_=34.40, *P*<0.001; Fig. 4). We also found that the interaction between temperature and humidity affected EHL/MHP (*F*_4,206_=49.40, *P*<0.001). Under dry conditions, EHL/MHP increased as temperatures increased (Fig. 4). Under humid conditions, EHL/MHP did not change as temperatures increased (Fig. 4). We found no evidence that mass (*P*=0.471) or sex (*P*=0.980) affected EHL/MHP.

### Biophysical simulations

**Fig. 5.**
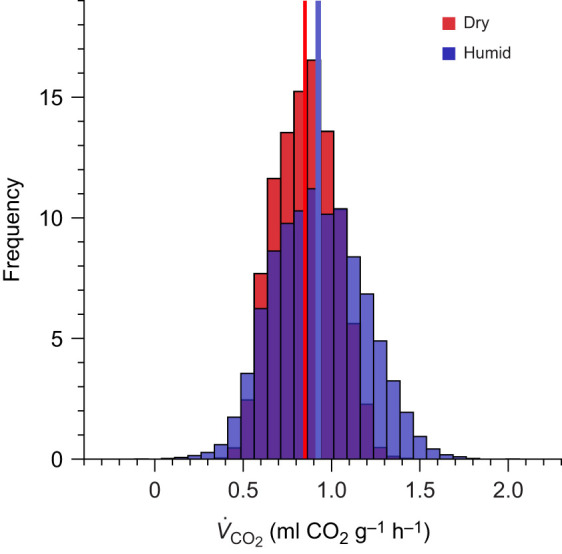
**Simulated rates of resting *V*_CO_2__ were lower under dry conditions (0% relative humidity; red) than humid conditions (60% relative humidity; blue) at our field site.** Biophysical simulations were run over local weather conditions during nine breeding seasons (2014–2023) and restricted to the range of temperatures in our laboratory experiments (15–32°C). The median rate of resting *V*_CO_2__ under dry conditions (0.860 ml CO_2_ g^−1^ h^−1^; red vertical line) was 7.6% lower than those under humid conditions (0.925 ml CO_2_ g^−1^ h^−1^; blue vertical line).

Despite the lack of significant differences in *V*_CO_2_ _or LCT between humid and dry conditions in our laboratory experiments, both values tended to be greater under humid conditions (Fig. 1). Parameterizing our biophysical model with these *V*_CO_2_ _and LCT values (and their associated variance) revealed that median rates of resting *V*_CO_2_ _in the field are 7.4±0.4% greater when using Scholander–Irving models of *V*_CO_2_ _under humid conditions compared with dry conditions (*P*<0.001; Fig. 5).

### Lab-to-field results

#### Body temperature

We found no evidence that average lab *T*_b_ (*P*=0.398) or its interaction with humidity (*P*=0.374) were associated with average field *T*_b_. Similarly, neither average field *T*_a_ (*P*=0.321) nor standard deviation in field *T*_a_ (*P*=0.591) were associated with average field *T*_b_. We also found no evidence that standard deviation in lab *T*_b_ (*P*=0.149), its interaction with humidity (*P*=0.603), or average field *T*_a_ (*P*=0.645) predicted standard deviation in field *T*_b_. However, we did find a nearly significant, negative association between standard deviation in field *T*_a_ and standard deviation in field *T*_b_ (*F*_1,10_=4.92, *P*=0.051). In other words, we found that *T*_b_ was less variable when *T*_a_ was more variable in the field.

#### Provisioning rate

Our provisioning rate models found that average field *T*_a_ (*F*_1,11_=6.30, *P*=0.050) and standard deviation in field *T*_a_ (*F*_1,11_=8.73, *P*=0.025) were negatively associated with provisioning rates, meaning that birds provisioned offspring at lower rates under warmer, more variable air temperatures. We did not find conclusive evidence that *V*_CO_2_ _within the TNZ measured in the lab was significantly associated with provisioning rates in the field (*P*=0.089), but we did find a marginally significant interaction between lab *V*_CO_2_ _within the TNZ and humidity (*F*_1,11_=4.03, *P*=0.069) in our provisioning rate model. Specifically, *V*_CO_2_ _within the TNZ measured under dry lab conditions was negatively correlated with field provisioning rates, whereas *V*_CO_2_ _within the TNZ measured under humid lab conditions was positively correlated with field provisioning rates (Fig. S2). We also found that individuals with greater temperature sensitivity of *V*_CO_2_ _in the lab provision offspring at higher rates in the field (*F*_1,11_=7.64, *P*=0.018; Fig. S3). We found no evidence that the interaction between temperature sensitivity of *V*_CO_2_ _and humidity was associated with field provisioning rate (*P*=0.281).

## DISCUSSION

Overall, we did not find statistically significant effects of humidity on tree swallow thermoregulatory energetics in our laboratory experiments. This may reflect a combination of relatively low sample sizes, higher variability of *V*_CO_2_ _under humid conditions, and relatively small statistical effects of humidity on *V*_CO_2__. Nevertheless, our biophysical model indicates that the small increases in LCT and *V*_CO_2_ _under humid conditions relative to dry conditions (Fig. 1) may generate meaningful increases in the energetic expenditure in the field. The median difference in simulated rates of resting *V*_CO_2_ _under humid versus dry conditions (7.4%) is comparable to the magnitude of differences in basal metabolic rate that affect fitness in field studies of birds and small mammals (Jackson et al., 2001; Larivée et al., 2010; Rønning et al., 2015). The higher *V*_CO_2_ _of tree swallows under cold, humid conditions may be an adaptive response to the high thermal conductivity (and thus high cooling power) of cold, humid air (Crow, 1988) or the high specific heat capacity of humid air (Denny, 1993). In support of this hypothesis, we found that tree swallows maintained slightly higher body temperatures under humid conditions compared with dry conditions, despite the greater conductance of cold, humid air. However, maintaining warmer body temperatures in the field comes at a cost of higher metabolic expenditure under conditions when the availability of flying insects (the primary prey of aerial insectivores such as tree swallows) is at its lowest (Garrett et al., 2022). High metabolic rates combined with low food availability likely explains the high levels of adult mortality (Weatherhead et al., 1985; Lombardo, 1986; Littrell, 1992) and reproductive failure (Cox et al., 2019; Garrett et al., 2022) observed in tree swallow populations during cold, wet weather. As tree swallow breeding phenology advances in response to warmer spring temperatures under climate change (Dunn and Winkler, 1999), individuals are increasingly likely to encounter periods of high metabolic demands and low food availability, which appears to be driving rapid population declines in tree swallows and possibly other aerial insectivores (Nebel et al., 2010; Shipley et al., 2020).

Interestingly, tree swallows appear to decouple the effects of humidity on evaporative water loss from *T*_b_ or metabolic demands. We found that EWL was higher under dry conditions than under humid conditions (Fig. 2A), similar to many studies on the effects of humidity (Chew and Dammann, 1961; Edwards and Haines, 1978; Webster and King, 1987; Powers, 1992; Klüg-Baerwald and Brigham, 2017). We also found that this effect is driven by the greater VPD of dry air compared with humid air, as illustrated by VPD-corrected EWL (Fig. 2B). Increased evaporative cooling at low temperatures under dry conditions should exacerbate heat loss (Edwards and Haines, 1978), potentially increasing the risk of hypothermia. If greater EWL resulted in heat loss, we would expect to find lower *T*_b_, a higher LCT, greater thermal sensitivity of *V*_CO_2__, and/or elevated *V*_CO_2_ _under dry conditions. We found no evidence for any of these predictions, like previous studies on some birds and mammals (Edwards and Haines, 1978; Webster and King, 1987; Powers, 1992). These data indicate that endotherms have the capacity to decouple EWL, *T*_b_ and metabolic rate, potentially through piloerection (which would maintain *T*_b_ with minimal energetic cost; Chaplin et al., 2014) or restricting cooling to integumentary or respiratory surfaces via vasoconstriction without affecting core body temperature (Zuluaga and Danner, 2023). Furthermore, EWL did not increase with warmer treatments (Fig. 2A), which also had higher evaporative demands (i.e. higher VPDs). Indeed, VPD-corrected EWL rates were lowest under warm temperatures (Fig. 2B). This could reflect active water loss regulation to minimize EWL under warm conditions with higher VPD (e.g. Cooper et al., 2020) or it could simply reflect greater metabolic rates (and associated increases in exhaled water vapor) under colder temperatures as metabolic demands increase. Regardless, our results and those of others suggest that EWL may not have large effects on *T*_b_ or metabolic rate at low temperatures, though additional work might focus on how birds regulate the thermoregulatory effects of increased water loss.

Our lab-to-field comparisons found mixed evidence that thermal physiology measured in the lab corresponds to thermoregulatory performance (or performance in general) in the field. For example, we found that individual variation in resting *T*_b_ in the lab was not correlated with active *T*_b_ in the field, for both average *T*_b_ and variability in *T*_b_. These results are surprising, given the high repeatability of field active body temperatures among individual tree swallows in previous work (Tapper et al., 2021). However, we also found that individuals with more thermally sensitive *V*_CO_2_ _below the TNZ in the lab provisioned their offspring at higher rates in the field than less thermally sensitive individuals. This may support the hypothesis that individuals with more plastic phenotypes have fitness advantages over less plastic individuals, especially in dynamic, fluctuating environmental conditions (Lande, 2014), which is characteristic of breeding season weather conditions at our study site. Finally, we found some evidence that measuring physiological traits under different humidity conditions in the lab altered the relationship between lab-based physiology measurements and field performance. Specifically, we found that *V*_CO_2_ _within the TNZ and provisioning rates are negatively correlated when *V*_CO_2_ _is measured under dry lab conditions, but positively correlated when *V*_CO_2_ _is measured under humid lab conditions. This may suggest that *V*_CO_2_ _within the TNZ measured under humid lab conditions is a more relevant measure of individuals’ physiological capacity to provision young under humid field conditions than *V*_CO_2_ _measured under dry lab conditions (Nilsson, 2002). Nonetheless, we found no effect of humidity in our other lab-to-field comparisons. Collectively, these results paint a mixed picture of whether lab-based physiology measurements are useful for understanding individual variation in the field, even when measured under more humid (and thus ecologically relevant) conditions. Future work that explores these relationships in other systems and focuses on increasing the ecological relevance of lab-based measurements would be extremely valuable for advancing our understanding of phenotype–performance relationships in physiological ecology.

Our work contributes to a growing body of literature indicating that, in some endotherms, humidity may have important thermoregulatory consequences. Interestingly, the thermoregulatory effects of humidity appear to vary tremendously across endotherms, both above the TNZ (Lasiewski et al., 1966; Webster and King, 1987; Powers, 1992; Gerson et al., 2014; van Dyk et al., 2019) and below it (Chew and Damman, 1961; Edwards and Haines, 1978; Webster and King, 1987; Powers, 1992; Cooper et al., 2020; Gilson et al., 2021). More work is needed to characterize these physiological responses and test their ecological consequences across a wider range of taxa, particularly given that global temperature and humidity patterns are projected to change over the next century (Trenberth et al., 2003). Like temperature, the change in humidity under future climate change is expected to be unevenly distributed across the globe (IPCC, 2023). Yet the physiological effects of humidity and uneven distribution of humidity are rarely included in models that forecast organismal responses to climate change (Jaworski and Hilszczański, 2013). Our work and that of others suggests that incorporating these effects may be necessary for accurately predicting the consequences of climate change for some endotherms.

## Supplementary Material

10.1242/jexbio.247357_sup1 Supplementary information
