# Supplementary material for: The effects of humidity on thermoregulatory physiology of a small songbird
Source: J Exp Biol. 2024 Jul 2;227(13):jeb247357. doi: 10.1242/jeb.247357 (PMC11418195; doi:10.1242/jeb.247357)
Supplement: Supplementary information [file jexbio-227-247357-s1.pdf]

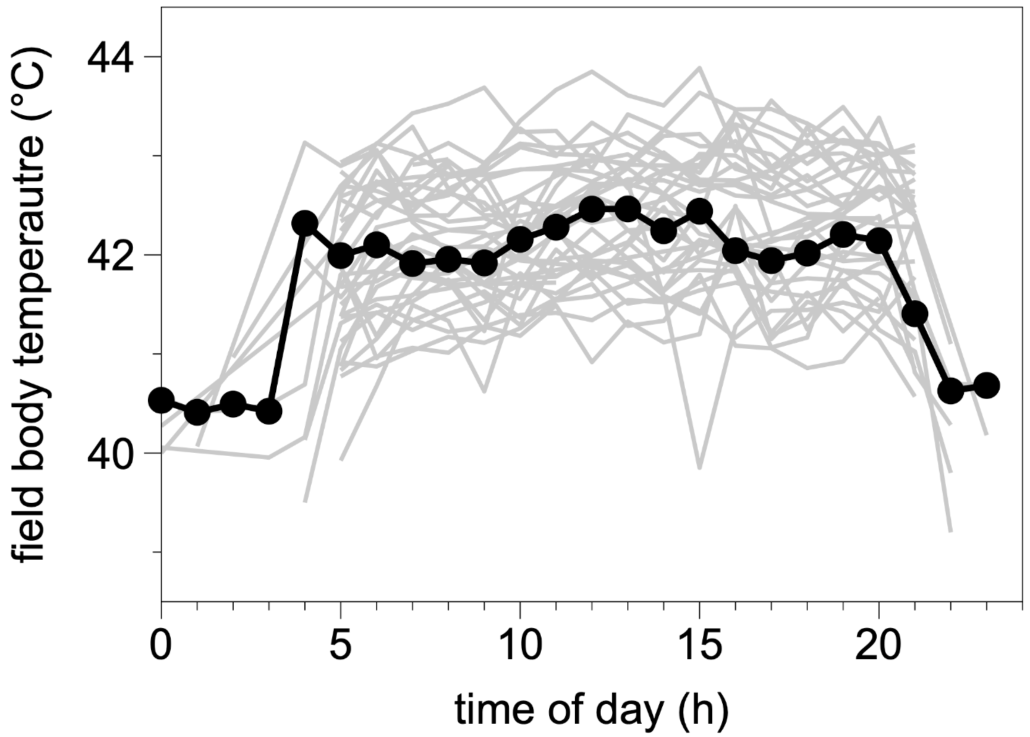

Fig. S1. Average field Tb of individual tree swallows (*n* = 17 adults) over the course of a 24-hour period based on PIT tag data. The black line denotes the average Tb across individuals, with each circle corresponding to the average Tb at a different hour of the day.

Journal of Experimental Biology • Supplementary information

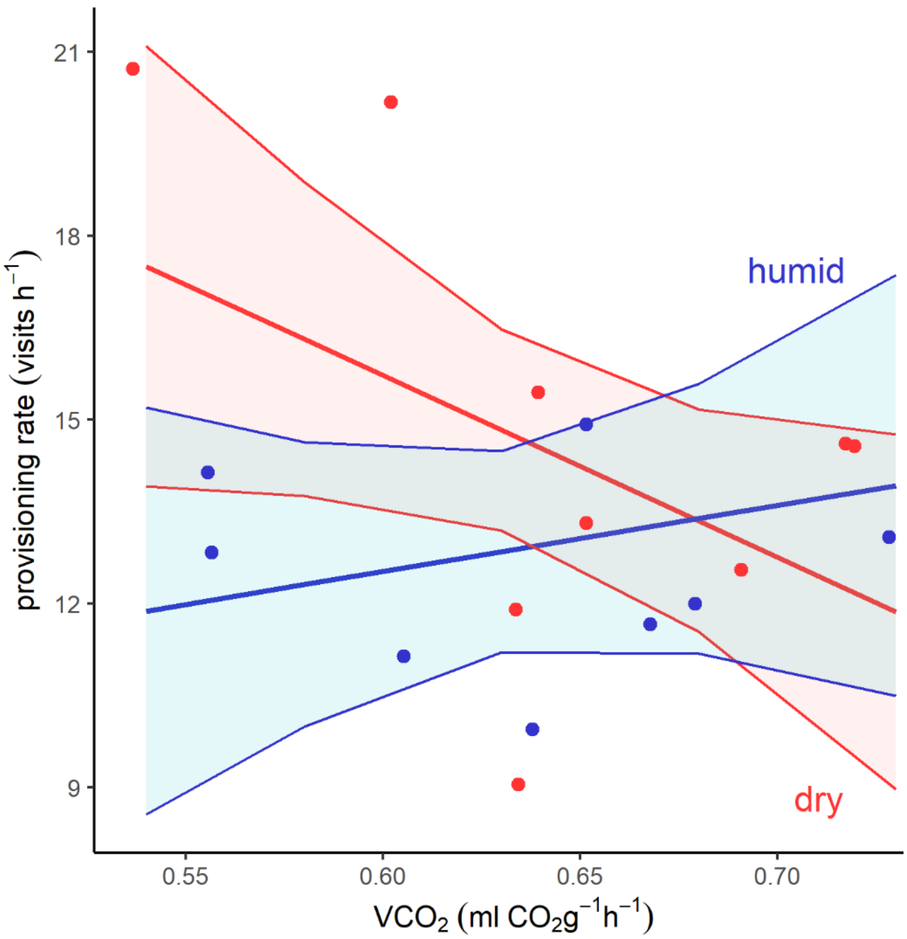

Fig. S2. Offspring provisioning rates in the field were negatively correlated with *V*CO_2 within the TNZ measured under dry lab conditions (red) but positively correlated with *V*CO2 within the TNZ measured under humid conditions (blue). *V*CO_2 was the average *V*CO2 within the TNZ (15-23ºC). Model predictions are plotted with the linear curves and shaded 95% confidence intervals. Circles are raw data from 17 adult tree swallows measured in the lab and the field.

Journal of Experimental Biology • Supplementary information

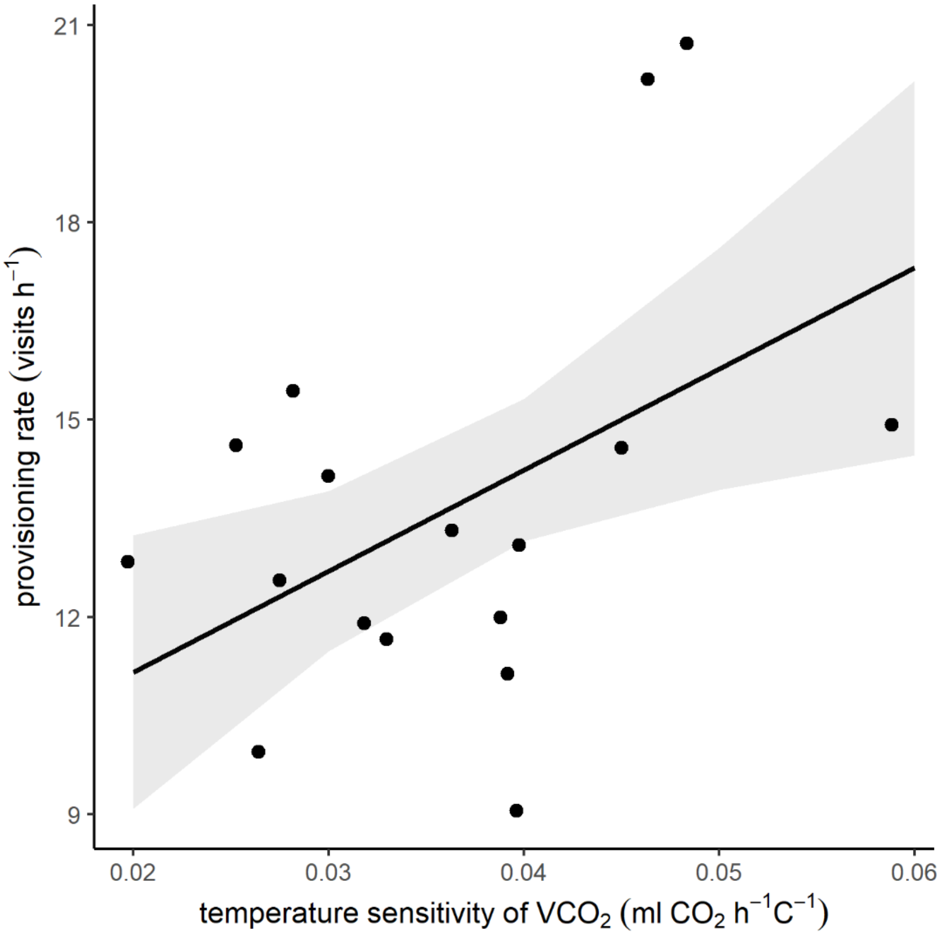

Fig. S3. Tree swallows with greater temperature sensitivity of $\dot{V}CO_2$ below the TNZ (as measured in the lab) provisioned their young at higher rates in the field. Temperature sensitivity of $\dot{V}CO_2$ is defined as the slope of the relationship between temperature and $\dot{V}CO_2$ below the TNZ (15-23ºC). For illustration purposes, we plotted the absolute slope of this relationship. Model predictions are plotted with the solid black line and shaded 95% confidence intervals. Black circles are raw data from 17 adult tree swallows measured in the lab and the field.

Journal of Experimental Biology • Supplementary information

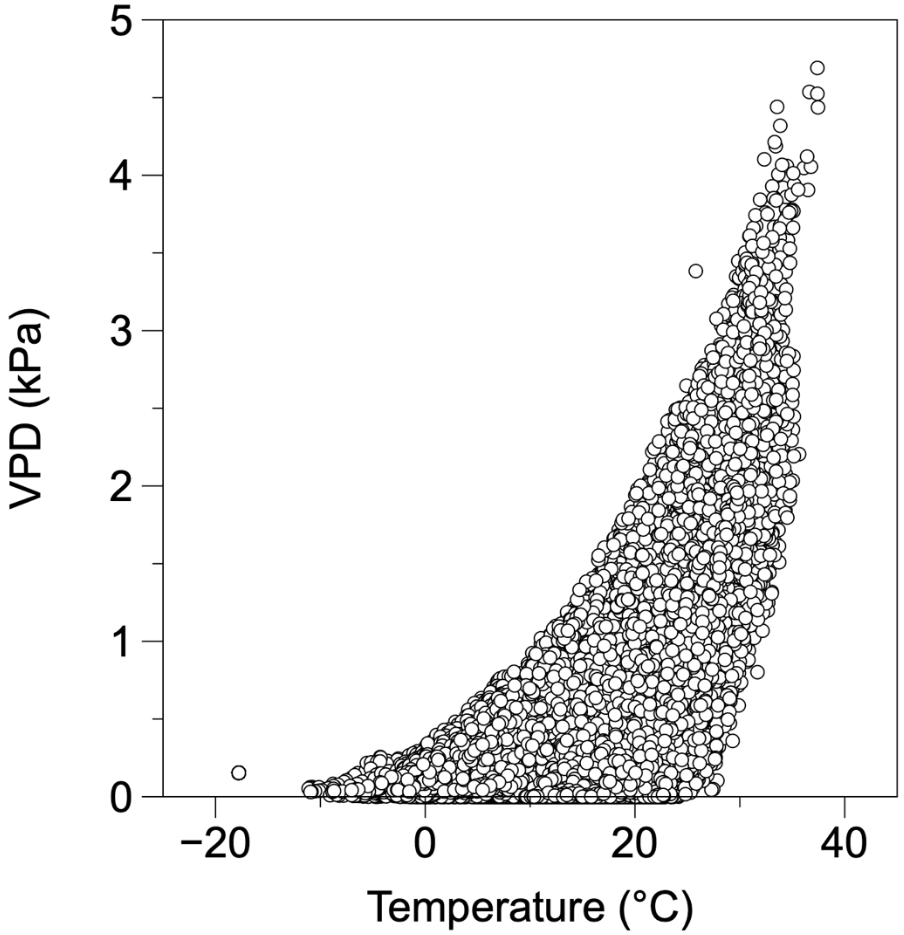

Fig. S4. The vapor pressure deficit (VPD; kPa) is positively and non-linearly correlated with temperature. The figure demonstrates that tree swallows experience a wide range of VPDs at nearly every air temperature that they experience.

Table S1. Absolute humidity (g/m^3; top row) and vapor pressure deficit (kPa; bottom row) of incurrent (Inc) and excurrent air (Exc) under different humidity-temperature combinations. The mean and standard deviation are reported for each humidity-temperature combination (note that incurrent absolute humidity for all dry trials at 23°C and 32°C was 0 g/m^3).

	**Dry (0% relative humidity)**	**Humid (60% relative humidity)**
**15°C**	*Inc:* 0.011 ± 0.038; *Exc:* 0.358 ± 0.126<br><br>*Inc:* 1.742 ± 0.019; *Exc:* 1.698 ± 0.021	*Inc:* 7.895 ± 0.142; *Exc:* 8.058 ± 0.154<br><br>*Inc:* 0.683 ± 0.025; *Exc:* 0.669 ± 0.027
**19°C**	*Inc:* 0.057 ± 0.079; *Exc:* 0.487 ± 0.171<br><br>*Inc:* 2.239 ± 0.038; *Exc:* 2.181 ± 0.053	*Inc:* 9.750 ± 0.448; *Exc:* 9.920 ± 0.433<br><br>*Inc:* 0.925 ± 0.193; *Exc:* 0.902 ± 0.071
**23°C**	*Inc:* 0; *Exc:* 0.376 ± 0.080<br><br>*Inc:* 2.918 ± 0.038; *Exc:* 2.867 ± 0.044	*Inc:* 11.946 ± 0.170; *Exc:* 12.085 ± 0.191<br><br>*Inc:* 1.283 ± 0.041; *Exc:* 1.278 ± 0.044
**30°C**	*Inc:* 0.018 ± 0.073; *Exc:* 0.397 ± 0.088<br><br>*Inc:* 4.398 ± 0.054; *Exc:* 4.350 ± 0.049	*Inc:* 17.691 ± 0.240; *Exc:* 17.824 ± 0.250<br><br>*Inc:* 1.926 ± 0.062; *Exc:* 1.884 ± 0.061
**32°C**	*Inc:* 0; *Exc:* 0.422 ± 0.067<br><br>*Inc:* 5.024 ± 0.045; *Exc:* 4.963 ± 0.046	*Inc:* 19.464 ± 0.575; *Exc:* 19.547 ± 0.601<br><br>*Inc:* 2.261 ± 0.109; *Exc:* 2.194 ± 0.104
